# The survival prediction of advanced colorectal cancer received neoadjuvant therapy—a study of SEER database

**DOI:** 10.1186/s12957-024-03458-7

**Published:** 2024-07-01

**Authors:** Zhuo Han, Haicheng Yang, Qing Qiao, Tao Wu, Xianli He, Nan Wang

**Affiliations:** Department of General Surgery, Tangdu Hospital, The Air Force Medical University, Xi’an, 710038 China

**Keywords:** CRC, SEER database, Neoadjuvant therapy, Survival, LODDS

## Abstract

**Purpose:**

The aim of study was to screen factors associated with the overall survival of colorectal cancer patients with lymph nodes metastasis who received neoadjuvant therapy and construct a nomogram model.

**Methods:**

All enrolled subjects of the SEER database were randomly assigned to the training and testing group in a ratio of 3:2. The patients of Tangdu Hospital were seemed as validation group. Univariate cox regression analysis, lasso regression and random forest survival were used to screen variables related to the survival of advanced CRC patients received neoadjuvant therapy in the training group. Area under curves were adopted to evaluate the 1,3,5-year prediction value of the optimal model in three cohorts. Calibration curves were drawn to observe the prediction accuracy of the nomogram model. Decision curve analysis was used to assess the potential clinical value of the nomogram model.

**Results:**

A total of 1833 subjects were enrolled in this study. After random allocation, 1055 cases of the SEER database served as the training group, 704 cases as the testing group and 74 patients from our center as the external validation group. Variables were screened by univariate cox regression used to construct a nomogram survival prediction model, including M, age, chemotherapy, CEA, perineural invasion, tumor size, LODDS, liver metastasis and radiation. The AUCs of the model for predicting 1-year OS in the training group, testing and validation group were 0.765 (0.703,0.827), 0.772 (0.697,0.847) and 0.742 (0.601,0.883), predicting 3-year OS were 0.761 (0.725,0.780), 0.742 (0.699,0.785), 0.733 (0.560,0.905) and 5-year OS were 0.742 (0.711,0.773), 0.746 (0.709,0.783), 0.838 (0.670,0.980), respectively. The calibration curves showed the difference between prediction probability of the model and the actual survival was not significant in three cohorts and the decision curve analysis revealed the practice clinical application value. And the prediction value of model was better for young CRC than older CRC patients.

**Conclusion:**

A nomogram model including LODDS for the prognosis of advanced CRC received neoadjuvant therapy was constructed and verified based on the SEER database and single center practice. The accuracy and potential clinical application value of the model performed well, and the model had better predictive value for EOCRC than LOCRC.

**Supplementary Information:**

The online version contains supplementary material available at 10.1186/s12957-024-03458-7.

## Introduction

The burden of colorectal cancer (CRC) on human life is increasing. The latest global cancer statistics data showed that CRC has become the third most common cancer, whose morbidity and mortality are rising year by year [1]. In 2017, a study proved that the diagnosis and treatment of CRC imposed a catastrophic financial burden on Chinese patients based on statistics from multiple hospitals in China [2]. Meanwhile, several epidemiological studies have found that although the overall incidence rate of CRC was relatively stable or even decreasing, stratified analyses suggested that the morbidity and mortality of young CRC patients were on the rise [3]. And clinicopathological characteristics studies have revealed that the prognosis of patients with early-onset CRC (EOCRC) is worse than late-onset CRC (LOCRC) patients [4]. Currently, surgery, chemotherapy and radiotherapy are still major clinical treatment measures of CRC patients, and the overall 5-year survival rate of Chinese CRC patients has increased from 47.2 to 56.9% from 2003 to 2015 [5]. However, the survival rate of early-stage patients at about 80%, but the advanced patients at less than 15% [6]. Although studies of targeted and immune drugs have been able to improve the survival of advanced patients to some extent, the status of their survival remains a concern.

Advanced CRC patients are at high risk of metastasis and recurrence, and several studies have confirmed that preoperative chemoradiotherapy or immunotherapy could downstage or stabilize tumors, shrinking of lesions facilitated radical surgical procedures, which in turn reduced the risk of recurrence. However, neoadjuvant chemoradiotherapy may be able to promote distant metastasis of tumors [7], and the benefit of long-term survival remains controversial [8–10]. In 2024, a retrospective study conducted by Tao Zhang et al. evaluated the safety and efficacy of apatinib combined with the XELOX regimen for neoadjuvant treatment of locally advanced CRC patients, the results found that the combination therapy achieved better objective remission and major pathologic remission rates and significantly prolonged the disease free survival (DFS) compared with XELOX alone [11]. Immunotherapy is superior to conventional or targeted therapy for unresectable advanced or metastatic CRC patients, and the NCCN (2021) guidelines recommend double-immunotherapy (Nivolumab combined with Ibritumomab or Pembrolizumab) as a new option for neoadjuvant treatment in patients with MSI-H/dMMR status [12, 13]. The fact that neoadjuvant therapy can provide patients with short-term benefits is indisputable, but long-term survival is still controversial. Therefore, predicting the long-term overall survival (OS) of advanced CRC patients received neoadjuvant therapy is essential. However, the constructive of survival prediction models for advanced CRC patients receiving neoadjuvant therapy are still lacking.

Intraoperative lymph node dissection and the number of positive lymph nodes is one of the most important factors affecting the prognosis of patients. The log odd of positive lymph node (LODDS) is a novel composite index calculated as log([NPLN + 0.5]/[NDLN-NPLN + 0.5]), which has been demonstrated to be a better predictor of predicting an individual’s survival status compared with the traditional N stage [14]. Qing-Wei Zhang et al. illustrated that LODDS was associated with the prognosis of CRC patients through the the Surveillance, Epidemiology, and End Results (SEER) database and international multicenter data [15]. Ben Huang et al. also found that LODDS was better than lymph nodes ratio (LNR) in predicting the prognosis of rectal cancer patients treated with preoperative radiotherapy [16], and the indicator performed well in EOCRC patients [17]. Therefore, this study included LODDS as an important predictor to construct a survival prediction model for advanced CRC patients who received neoadjuvant therapy.

Therefore, this study would focus on the survival prediction of advanced CRC patients with lymph node metastasis and who received neoadjuvant therapy. The purposes of the study were to screen the influencing factors related to survival and construct a nomogram model by using various statistical methods in the SEER database. Additionally, this study also explored the predictive value of the model for the survival of both EOCRC and LOCRC.

## Results

### Basic characteristics

**Fig. 1:**
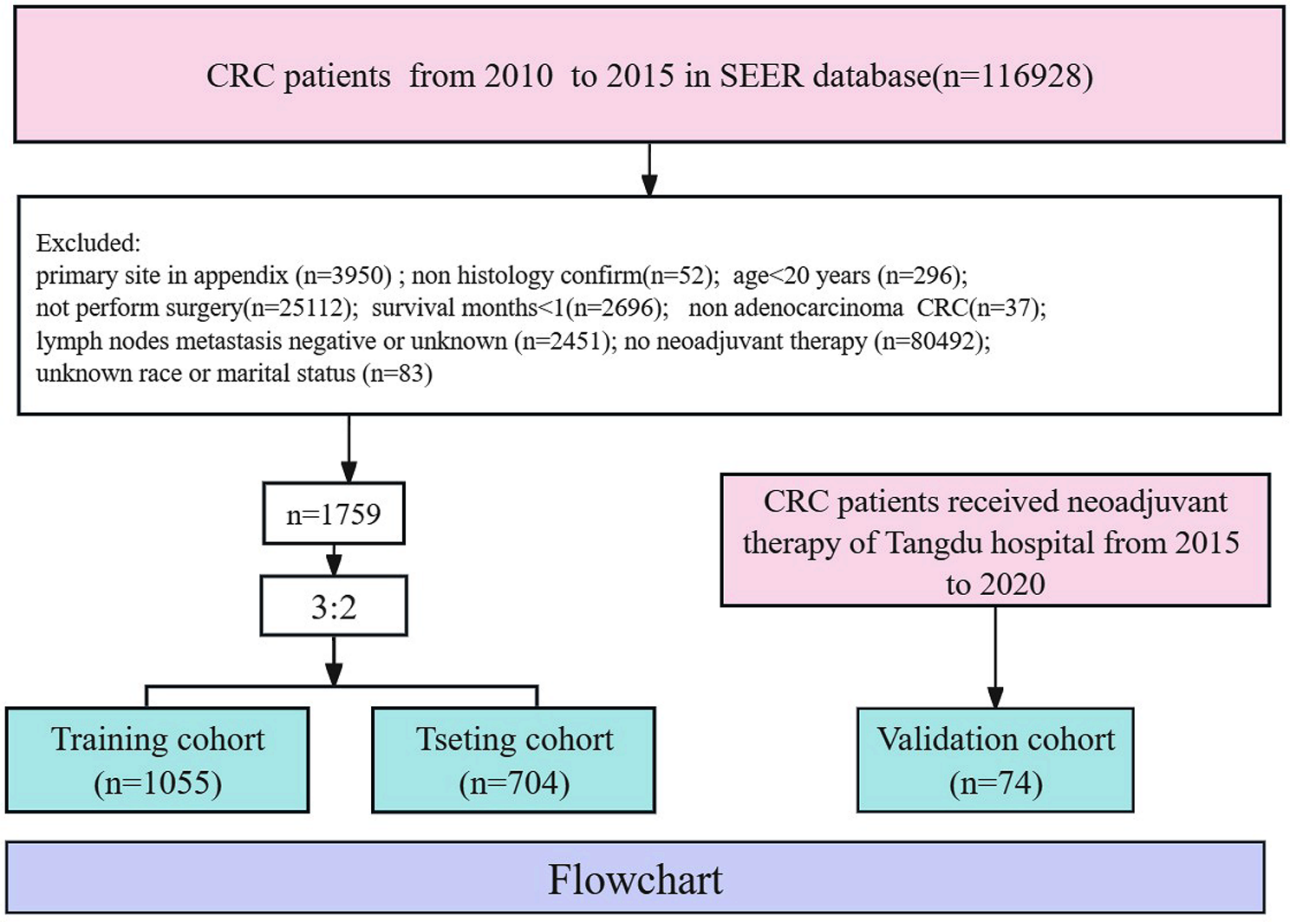
The detailed flowchart of the study

Figure 1 showed that a total of 1833 study subjects were enrolled to conduct this study, including 1759 cases in the SEER database and 74 cases in our center. The SEER dataset was randomly assigned in a 3:2 ratio, 1055 cases were used as the training group and the testing group was 704 cases. The 74 patients in our center served as the validation group. As the results presented in Table 1, there was no significant statistical difference were observed in clinical features between the training group and the testing group, except for gender. The Table 2 showed the clinical characteristics of patients in Tangdu hospital. The proportion of LOCRC patients was near to 80% and the tumor location of 75% patients was rectal. The pathology type of most patients was moderately differentiated. And more patients received radiotherapy and chemotherapy, with low levels of LODDS and LNR as the main characteristics.

**Table 1:** Characteristics of training group and testing group

Characteristics, *n* (%)	Training group (*N* = 1055)	Testing group (*N* = 704)	χ^2^	*P*
**Age**			0.101	0.751
<50	230(21.8)	149(21.2)		
≥50	825(78.2)	555(78.8)		
**Race**			0.609	0.737
white	833(79.0)	565(80.3)		
black	75(7.1)	44(6.2)		
other	147(13.9)	95(13.5)		
**Gender**			6.085	0.014
male	634(60.1)	464(65.9)		
female	421(30.9)	240(34.1)		
**Marital status**			0.108	0.947
married	657(62.3)	433(61.5)		
unmarried/single	196(18.6)	133(18.9)		
divorced/separated/widowed	202(19.1)	138(19.6)		
**History of tumor**			0.008	0.930
one primary only	844(80.0)	562(79.8)		
other	211(20.0)	142(20.2)		
**Primary site**			0.971	0.615
right colon	76(7.2)	46(6.5)		
left colon	179(17.0)	131(18.6)		
rectum	800(75.8)	527(74.9)		
**Differentiation**			2.766	0.429
well	53(5.0)	35(5.0)		
moderately	693(65.7)	483(68.6)		
poorly/undifferentiation	200(19.0)	112(15.9)		
unknown	109(10.3)	74(10.5)		
**T stage**			0.527	0.769
T_x_-T_2_	120(11.4)	87(12.4)		
T_3_	763(72.3)	508(72.2)		
T_4_	172(16.3)	109(15.4)		
**N stage**			0.694	0.405
N_1_	774(73.4)	529(75.1)		
N_2_	281(26.6)	175(24.9)		
**M stage**			0.347	0.556
M_0_	788(74.7)	517(73.4)		
M_1_	267(25.3)	187(26.6)		
**Radiation sequence**			1.351	0.717
both	30(2.8)	26(3.7)		
preoperative	803(76.1)	530(75.3)		
postoperative	24(2.3)	19(2.7)		
unknown	198(18.8)	129(18.3)		
**Radiotherapy**			0.055	0.815
yes	857(81.2)	575(81.7)		
no/unknown	198(18.8)	129(18.3)		
**Chemotherapy**			3.732	0.053
yes	1036(98.2)	699(99.3)		
no/unknown	19(1.8)	5(0.7)		
**Time of diagnosis to treatment**			4.730	0.094
0	193(18.3)	152(21.6)		
1 month	615(58.3)	412(58.5)		
>1 month	247(23.4)	140(19.9)		
**CEA**			1.776	0.411
positive	460(43.6)	306(43.5)		
negative	366(34.7)	228(32.4)		
unknown	229(21.7)	170(24.1)		
**Perineural invasion**			3.176	0.204
positive	213(20.2)	129(18.3)		
negative	745(70.6)	523(74.3)		
unknown	97(9.2)	52(7.4)		
**Tumor size**			1.118	0.572
≤4 cm	426(40.4)	282(40.1)		
>4 cm	500(47.4)	324(46.0)		
unknown	129(12.2)	98(13.9)		
**Examined lymph nodes**			0.744	0.388
≥15	594(56.3)	411(58.4)		
<15	461(43.7)	293(41.6)		
**LODDS**			0.745	0.689
<-0.59	629(59.6)	418(59.4)		
-0.59 ≤ LODDS≤-0.17	253(24.0)	179(25.4)		
>-0.17	173(16.4)	107(15.2)		
**LNR**			2.011	0.367
<0.14	506(48.0)	322(45.7)		
0.14 ≤ LNR ≤ 0.42	395(37.4)	287(40.8)		
>0.42	154(14.6)	95(13.5)		
**Bone metastasis**			0.054	0.816
yes	5(0.5)	2(0.3)		
no/unknown	1050(99.5)	702(99.7)		
**Liver metastasis**			1.431	0.232
yes	206(19.5)	154(21.9)		
no/unknown	849(80.5)	550(78.1)		
**Lung metastasis**			4.682	0.030
yes	36(3.4)	39(5.5)		
no/unknown	1019(96.6)	665(94.5)		

**Table 2:** Characteristics of validation group

Characteristics, *n* (%)	Validation group (*N* = 74)
**Age**	
<50	26(35.1)
≥50	48(64.9)
**M stage**	
M_0_	48(64.9)
M_1_	26(35.1)
**Radiotherapy**	
yes	25(33.8)
no/unknown	49(66.2)
**Chemotherapy**	
yes	71(95.9)
no/unknown	3(4.1)
**CEA**	
positive	35(47.3)
negative	39(52.7)
**Perineural invasion**	
positive	18(24.3)
negative	56(75.7)
**Tumor size**	
≤4 cm	46(62.2)
>4 cm	28(37.8)
**Liver metastasis**	
yes	20(27.0)
no/unknown	54(73.0)
**LODDS**	
<-0.59	38(51.4)
-0.59 ≤ LODDS≤-0.17	20(27.0)
>-0.17	16(21.6)

### Screening variables associated with survival in training group

**Fig. 2:**
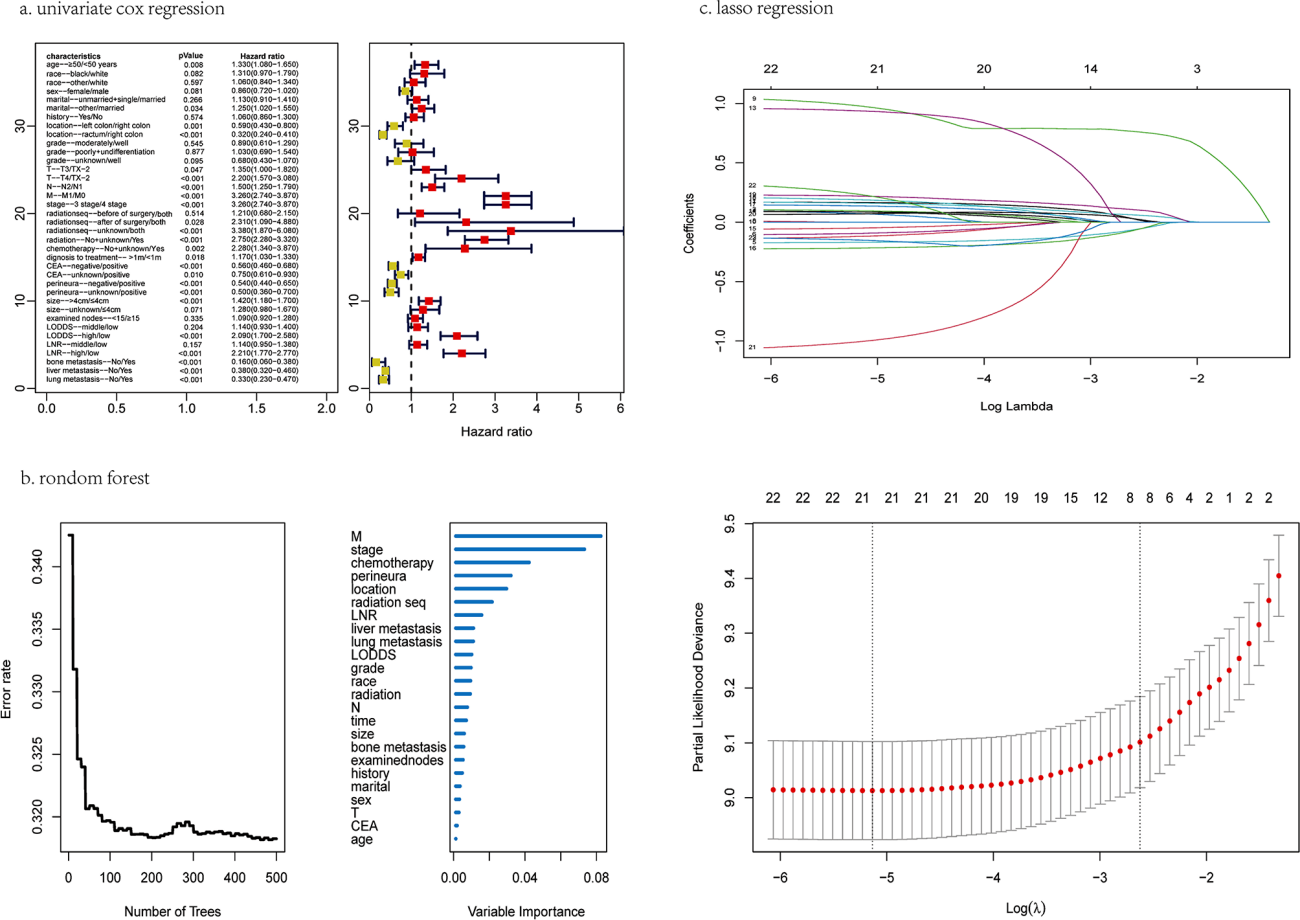
Screening variables associated with prognosis of advanced CRC received neoadjuvant therapy in the training group. (**a**) univariate cox regression, (**b**) randomForest survival (RFS), (**c**) lasso regression

Firstly, univariate cox regression, lasso regression and random forest were used to screen factors associated with OS of advanced CRC treated with neoadjuvant therapy, and the results were shown in Fig. 2. Univariate cox regression showed that age, marital status, location, T, N, M, stage, radiation sequence, radiotherapy, chemotherapy, time of diagnosis to treatment, CEA, perineural invasion, tumor size, LODDS, LNR and bone/liver/lung metastasis were associated with OS. The variables were selected through lasso regression, including location, T, M, stage, radiation sequence, perineural invasion, LODDS and LNR. The RFS results were ranked according to the importance of the variables, and some variables with relative importance greater than 12% were selected as candidates. Next, M, stage, location, chemotherapy, perineural invasion, radiation sequence, LNR, liver/lung metastasis and LODDS were chosen.

**Fig. 3:**
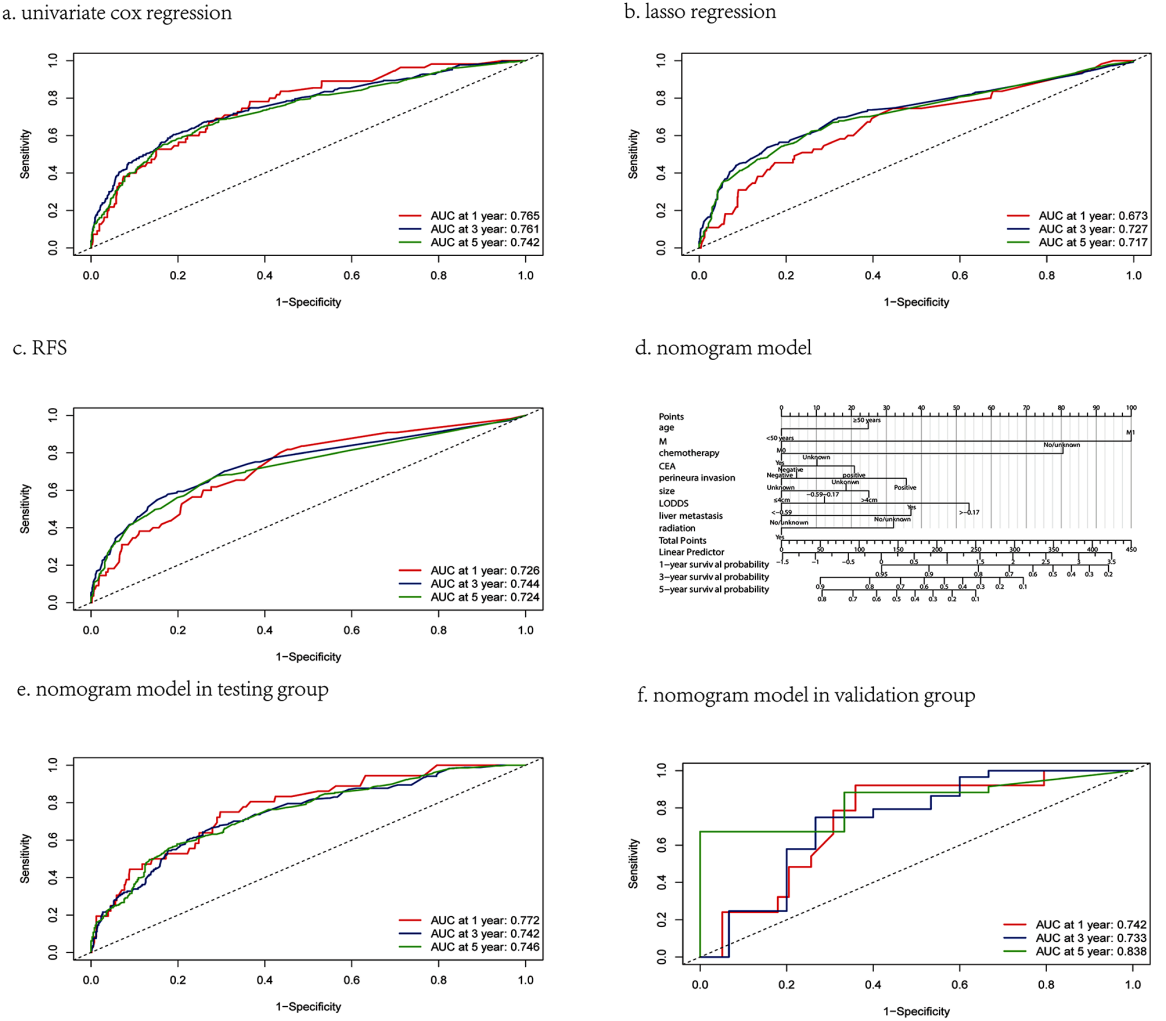
**a-c**. Compare 1,3,5-year prediction value of three models for advanced CRC in the training group (a. univariate cox regression, b. lasso regression, c. RFS). **d**. nomogram survival prediction model for advanced CRC received neoadjuvant therapy in the training group. **e-f**. The verified of model in the testing and validation group

Secondly, stepwise multivariate cox regression was used for further screening (Table S1-S3). The variables screened by univariate cox regression included M, age, chemotherapy, CEA, perineural invasion, tumor size, LODDS, liver metastasis and radiation, corresponding to an AIC and C-index of 7072.72 and 0.695, respectively. However, only five variables screened by the lasso regression were retained, including location, T, M, perineural invasion and LNR, corresponding to an AIC and C-index of 7099.68 and 0.67, respectively. The variables screened by the RFS were M, chemotherapy, perineural invasion, location, LNR and liver metastasis, which corresponded to an AIC and C-index of 7090.51 and 0.676, respectively. Finally, we found that the 1-year, 3-year and 5-year AUCs of the univariate cox regression were 0.765, 0.761 and 0.742, respectively, while the corresponding AUCs of lasso and RFS were 0.673, 0.727, 0.717 and 0.726, 0.744, 0.724, respectively (Fig. 3a-c). Considering the three evaluation indexes of the AIC, C-index and AUCs, we finally chose the variables screened by the univariate cox regression to construct the survival prediction model.

### Construction and validation of the nomogram model

A nomogram survival prediction model was constructed for advanced CRC patients treated with neoadjuvant therapy based on variables screened by univariate cox regression (Fig. 3d). A total score was calculated based on each patient’s clinical characteristic score, and their corresponding 1, 3 and 5-year survival rates were obtained. The study assessed the predictive ability of the model by AUCs. The results of the study (Fig. 3a, e and f) showed that the AUCs of the model for predicting 1-year OS in the training group, testing and validation group were 0.765 (0.703,0.827), 0.772 (0.697,0.847) and 0.742 (0.601,0.883), respectively, the AUCs for predicting 3-year OS was 0.761 (0.725,0.780), 0.742 (0.699,0.785), 0.733 (0.560,0.905) and AUCs for predicting 5-year OS were 0.742 (0.711,0.773), 0.746 (0.709,0.783), 0.838 (0.670,0.980), respectively. The predicted AUCs of the model were all above 0.70, so the model was considered to have some predictive value for OS in patients with advanced CRC treated with neoadjuvant therapy.

**Fig. 4:**
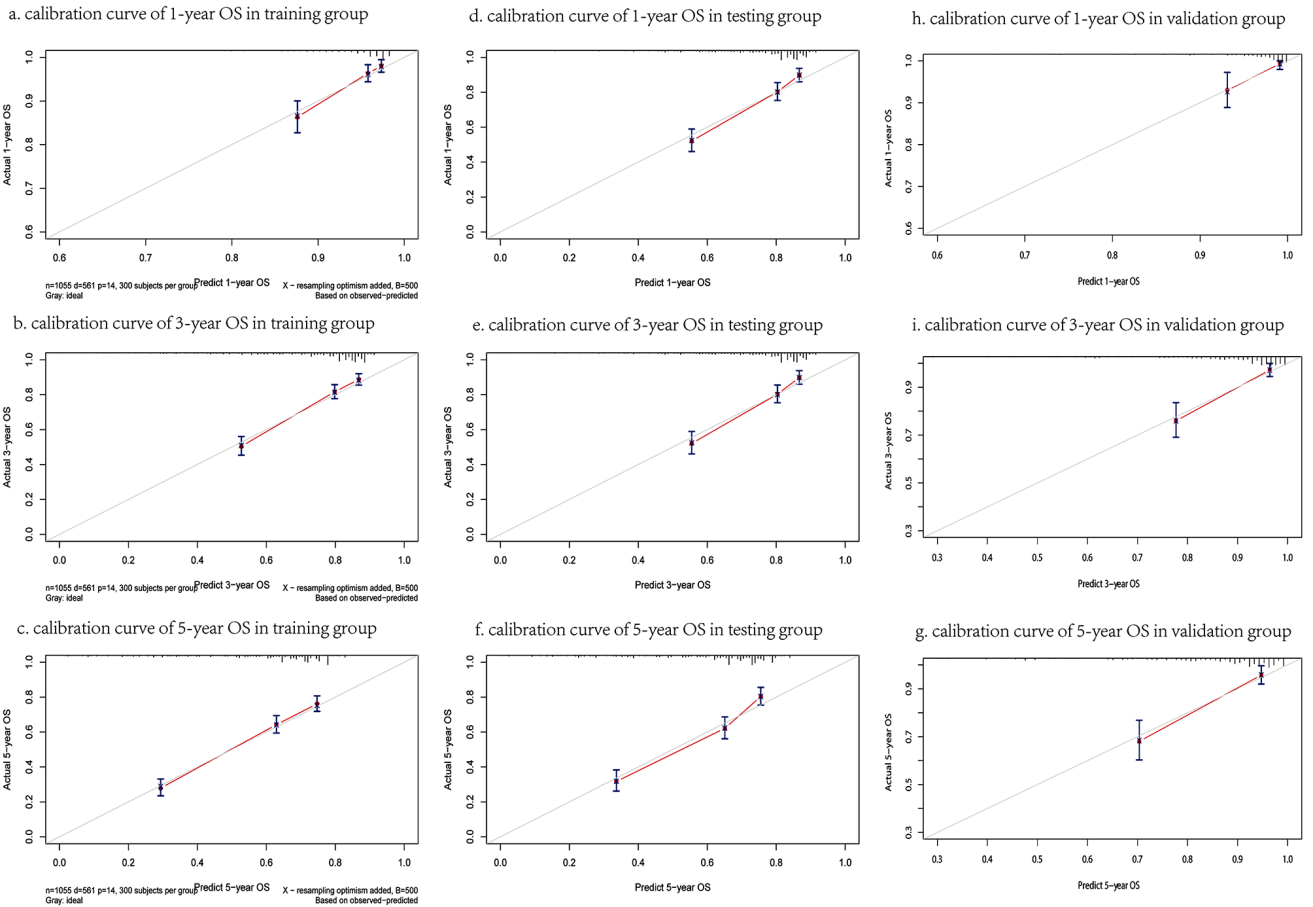
The figure showed the prediction accuracy of the nomogram model in the training, testing and validation group through calibration curve

**Fig. 5:**
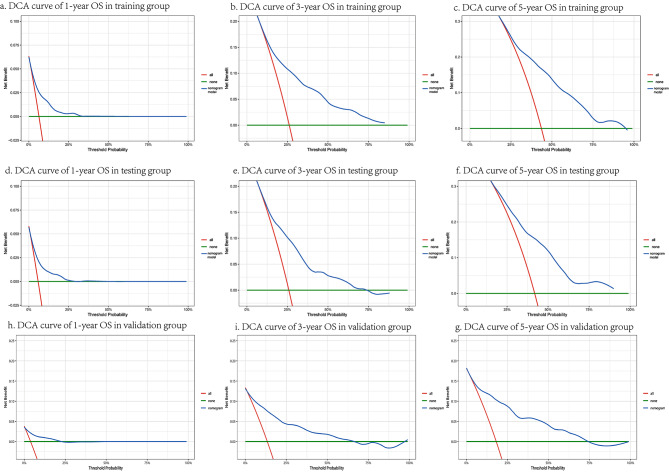
The figure evaluated the actual clinical application value of the nomogram model by using the DCA curve in the training, testing and validation group

Figure 4 demonstrated the accuracy of the model in the training, testing and validation groups through the calibration curves. The fit of the red line to the 45° calibration line illustrated the fit between the predicted results and the real results. We also calculated the C-index of the calibration curves. The study found the corrected C-index of the 1,3,5 years-calibration curves in the training group were 0.6862,0.6860 and 0.6869, respectively. And the corrected C-index of the 1,3,5 years-calibration curves in the testing and validation group were 0.6967,0.6964, 0.6943 and 0.6979,0.6967,0.6951, respectively. The results indicated the nomogram model has certain prediction ability. DCA curve presented that the difference between the predicted results and the real survival status was not significant. In addition, the DCA curve also was used to assess the practical clinical application value of the model, and the corresponding results were displayed in Fig. 5.

Subsequently, this study also evaluated the predictive value of the model in different N stages and LODDS grades (Table 3). The results suggested that the model had a higher predictive value in predicting 1-year and 3-year OS in patients with N2 compared with N1, and the predictive value of the model reached 0.862 (0.801,0.923) in predicting 1-year OS in patients with N2a. For patients with different levels of LODDS, the model had a higher predictive value for 1-year OS (0.729 (0.644,0.814)) for patients with low levels of LODDS, but a higher predictive value for 3-year and 5-year OS for patients with high levels of LODDS. The results were shown in supplement figure S1and figure S2.

**Table 3:** The prediction value of nomogram model in subgroups of N stage and LODDS

subgroup	*N*	Prediction value in 1-year OS	Prediction value in 3-year OS	Prediction value in 5-year OS
**N stage**				
N1a	565	0.713(0.613,0.814)	0.742(0.690,0.795)	0.750(0.707,0.792)
N1b	478	0.690(0.587,0.792)	0.694(0.637,0.751)	0.698(0.649,0.748)
N2a	469	0.862(0.801,0.923)	0.746(0.669,0.824)	0.695(0.626,0.764)
N2b	183	0.750(0.640,0.860)	0.791(0.725,0.857)	0.747(0.670,0.825)
**LODDS**				
low	1047	0.729(0.644,0.814)	0.713(0.673,0.754)	0.715(0.682,0.749)
middle	432	0.721(0.630,0.812)	0.724(0.668,0.781)	0.719(0.670,0.768)
high	557	0.719(0.628,0.810)	0.780(0.725,0.836)	0.766(0.710,0.822)

### Prediction of nomogram model for EOCRC and LOCRC

**Fig. 6:**
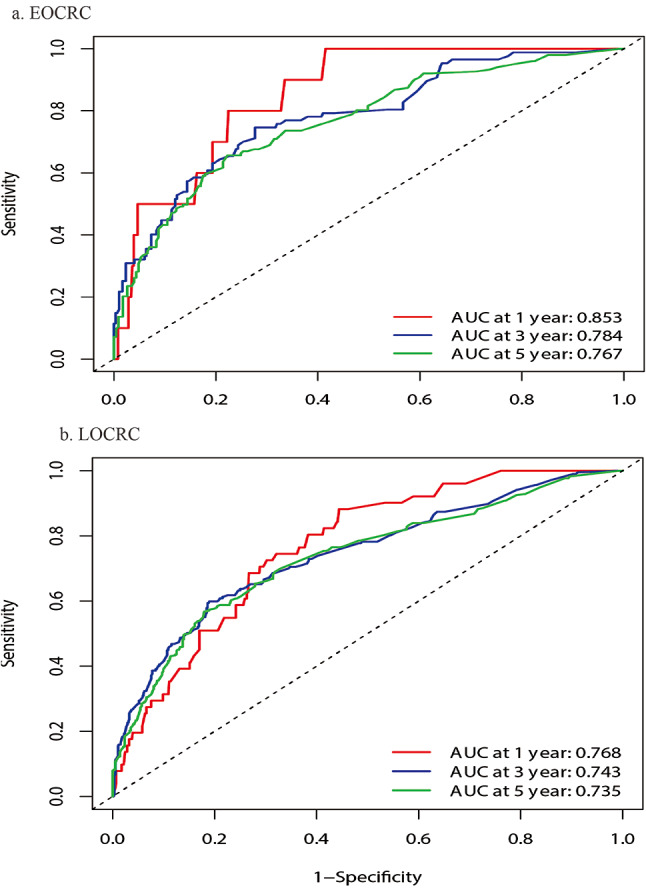
The figure showed the prediction value of the nomogram model for EOCRC and LOCRC received neoadjuvant therapy

This study also explored the predictive value of the established nomogram model for EOCRC and LOCRC. A total of 1833 patients from the SEER database and Tangdu hospital were included in the study, of which EOCRC and LOCRC patients were 405 and 1428, respectively. Considering the large difference in sample size between the two groups, PSM was conducted to balance the difference. Matching variables included gender, tumor location and stage, the caliper value was set as 0.01. Finally, 405 cases EOCRC patients and 810 cases LOCRC patients were included after matching. Figure 6 showed that the 1, 3 and 5-year OS of the model for patients with EOCRC and LOCRC received neoadjuvant therapy were 0.853 (0.766,0.939), 0.784 (0.728,0.839), 0.767 (0.718,0.815) and 0.768 (0.711,0.825), 0.743 (0.702,0.784), 0.735 (0.699,0.770).

## Discussion

In recent years, studies of the short-term benefits of neoadjuvant therapy have made it become the standard therapy for advanced CRC with lymph node positive. However, its effect on long-term survival remained controversial [18, 19]. And it has been found that neoadjuvant therapy altered body composition, which maybe influence the prognostic factors [20, 21], therefore, it was essential to study the long-term survival of patients and their prognostic factors after neoadjuvant therapy.

In this study, we screened factors associated with OS of advanced CRC patients received neoadjuvant therapy through the SEER database, and a nomogram model was constructed and validated. The results found that the model performed good predictive value for the survival of advanced CRC patients after neoadjuvant therapy. And the subgroup analyses indicated that the model has a high predictive value for N2 stage and high-level LODDS patients. The more important point was that the model has a greater predictive ability for survival in EOCRC than LOCRC patients.

Univariate cox regression, lasso regression and random forest were used to screen the variables, and the variables were selected furtherly through stepwise multivariate regression. The optimal combination of variables was evaluated by AUC value and C index together, including M, age, chemotherapy, CEA, perineural invasion, tumor size, LODDS, bone/liver metastasis and radiation. A study by Wei et al. in 2020 identified vascular cancer thrombus, tumor deposition, N stage, BMI, perineural invasion, postoperative CEA and distance of anal verge from the dentate line as influencing factors for OS in locally advanced rectal cancer undergoing neoadjuvant therapy [22]. And the team of Zhifang Zheng also demonstrated that the maximum diameter of the tumor, the distance from the anal verge, the preoperative CA19-9 level, ypTNM stage and perineural invasion were independent predictors of OS in patients with locally advanced CRC [23]. While, these studies lacked validation from external data. And in this study, we also found that the perineural invasion, CEA and tumor size were risk factors associated with OS after screening comprehensively potential variables, which were consistent with previous studies. At the same time, we also considered the prognostic role of the LODDS in CRC. Therefore, we concluded that age>50 years, positive CEA and perineural invasion, maximum tumor diameter > 4 cm, high level of LODDS, as well as the absence of any chemoradiotherapy during the treatment period and distant metastasis resulted in poor survival.

In addition, we evaluated the model in testing and validation group and the results showed that the model performed well with AUCs of 0.772 (0.697,0.847), 0.742 (0.601,0.883) for predicting 1-year OS, AUCs of 0.742 (0.699,0.785), 0.733 (0.560,0.905) for 3-year OS and AUCs of 0.746 (0.709,0.783), 0.838 (0.670,0.980) for 5-year OS, respectively. Then, we also assessed the prediction accuracy and clinical application value of the model, and the results showed that the model had good accuracy as well as certain clinical application value. More importantly, the study also compared the predictive value of the model in different N stage and LODDS subgroups. We found the predictive value of the model was better for N2 patients than N1, the predictive value of the 1-year OS was higher for low-level LODDS patients, but the predictive values of the 3- and 5-year OS were better in high level of LODDS patients, which maybe indicated that the model had a better long-term survival prediction for patients with high levels of lymph node involvement. However, the stability of the predictive value of LODDS was better than N stage, which may be because the index took into account the total number of lymph nodes removed, the number of positive lymph nodes and the number of negative lymph nodes, and previous studies have also found the LODDS was better than the N stage in predicting the prognosis [16, 24]. In 2022, Yanfei Lin also constructed a prognostic model based on the SEER database for patients with stage II/III rectal cancer who underwent neoadjuvant radiotherapy followed by surgical resection, and the AUC of the model for the 2, 3, and 5-year OS in the training and internal validation group were 0.736, 0.720, 0.688 and 0.691, 0.696, 0.694, respectively [25]. Compared with this model, the model constructed in our study has a higher survival prediction value for stage III-IV patients and has been verified in an external validation group. Meanwhile, the model also explored the ability to discriminate EOCRC and LOCRC and the results proved that the predictive value of the model for EOCRC was higher than LOCRC.

One of the strengths of this study was the selection of advanced CRC patients with positive lymph node metastases undergoing neoadjuvant therapy as the study subjects, which was necessary for the prediction of long-term survival of these patients who have poor prognosis and could benefit from neoadjuvant therapy in the short term [26, 27]. Second, LODDS, LNR and the total number of lymph node dissection were all enrolled as candidate variables in this study, but finally LODDS was screened and included in the model, which confirmed that this index can better reflect the status of the lymph nodes than other parameters [16, 28, 29]. Finally, the study screened the variables through various methods, and the constructed model was verified in both internal and external validation groups. The results of subgroup analyses also showed that the model had a high predictive value for EOCRC patients and the predictive value of the model was overall more stable in different LODDS subgroups than the N stage. However, this study still has some shortcomings. First, the variables included in the study only were some clinical characteristics, while variables such as BMI, frequency and regimen of neoadjuvant therapy, response after neoadjuvant therapy, economic, social factors, lifestyle habits and genetics were not included. Second, since fewer patients underwent neoadjuvant therapy in our center during the same period of time, the sample size of the external validation dataset included in this study is small. In addition, a retrospective study design may cause information bias to a certain degree.

## Methods

### Participants

Detailed information of patients was obtained from the SEER database containing 17 registries and the CRC specialized database of the Department of Gastrointestinal Surgery in Tang Du Hospital. Patients diagnosed with CRC in 2010–2015 in the SEER database were considered for inclusion in the study, and the patients were excluded if met the exclusion criteria of ① not performed surgery due to some reasons; ② survival time < 1 month; ③ age < 20 years; ④primary site in the appendix; ⑤ diagnosis not confirmed by pathology; ⑥no neoadjuvant therapy; ⑦ lymph node metastasis negative or metastasis unknown; ⑧ non-adenocarcinoma; ⑨ patients with unknown race or marital status. Ultimately, a total of 1,759 patients were enrolled in the study. All study subjects were randomly assigned into the training and testing group according to 3:2 ratio, and the detailed screening process was demonstrated in Fig. 1. 74 patients from our center’s database who met the inclusion exclusion criteria were used as the external validation group.

### Variables and outcome

The extracted variables included patient ID, age, race, year of diagnosis, marital status, sequence number, site recode, histology, grade, diagnostic confirmation, Derived EOD 2018 T (2018+), Derived EOD 2018 N (2018+), Derived EOD 2018 M(2018+), Derived EOD 2018 Stage Group (2018+), RX Summ–Surg/Rad Seq, Reason no cancer-directed surgery, Radiation recode, Chemotherapy recode (yes, no/unk), Months from diagnosis to treatment, CEA Pretreatment Interpretation Recode (2010+), Perineural Invasion Recode (2010+), CS tumor size (2004–2015), Regional nodes examined (1988+), Regional nodes positive (1988+), SEER Combined Mets at DX-bone (2010+), SEER Combined Mets at DX-liver (2010+), SEER Combined Mets at DX-lung (2010+), CS Tumor Size/Ext Eval (2004–2015), CS Reg Node Eval (2004–2015), Vital status recode (study cutoff used) and Survival months. “CS Tumor Size/Ext Eval (2004–2015)” and “CS Reg Node Eval (2004–2015)” were used to identify patients undergoing neoadjuvant therapy before surgery.

### Statistical analysis

All data analysis and drawing in the study were performed using R software (4.3.2 version). Regional nodes examined (1988+) and regional nodes positive (1988+) were used to compute LODDS and LNR. X-tile software was used to determine the optimal cut-off values for LODDS and LNR, which were converted into rank variables for subsequent analyses. The results showed that the optimal cutoff values for LODDS and LNR were − 0.59, -0.17 and 0.14, 0.42, respectively. LODDS was categorized into three groups: LODDS<0.59(low), -0.59 ≤ LODDS≤-0.17 (medium), LODDS>-0.17 (high). Similarly, LNR also was categorized into 3 groups: LNR< 0.14(low), 0.14 ≤ LNR ≤ 0.42(medium), LODDS > 0.42(high). Categorical variables were statistically described by n (%), and *P*<0.05 was considered a statistically significant difference.

First, the final included SEER data were divided into training and testing groups in a ratio of 3:2, our center data served as the validation group. Second, univariate cox regression analysis (survival package), last absolute shrinkage and selection operator (lasso) regression (glmnet package) and random forest (randomForest SRC package) were used to screen variables related to OS in the training group, and the selected variables were further analyzed by stepwise multivariate cox regression. Next, the timeROC package was used to plot receiver operating characteristic (ROC) curves of three models, and the optimal combination of the variables was selected to build model by comparing the AIC, C-index and AUC. Finally, the constructed nomogram model was validated in testing and validation groups, and the AUC was used to assess the predictive value of the model in the three datasets. The calibration curves were adopted to evaluate the predictive accuracy of the model, and the Decision Curve Analysis (DCA) (dcurves package) was used to assess the potential clinical value of the model.

Exploring the survival predictive value of the nomogram model for EOCRC and LOCRC patients, propensity score matching (PSM) was used to adjust for differences in baseline characteristics between patients with early- and late-onset CRC.

## Conclusion

In this study, we constructed and validated a survival prediction model of advanced CRC patients who received neoadjuvant therapy based on the SEER database and our center. The accuracy and potential clinical application value of the model performed well, and the model had better predictive value for EOCRC than LOCRC.

### Electronic supplementary material

Below is the link to the electronic supplementary material.

Supplementary Material 1

Supplementary Material 2

Supplementary Material 3

## Data Availability

More detailed data would be available if you request, please do not hesitate to contact the corresponding author.
