# Supplementary figures and images for: The survival prediction of advanced colorectal cancer received neoadjuvant therapy—a study of SEER database
Source: World J Surg Oncol. 2024 Jul 1;22:175. doi: 10.1186/s12957-024-03458-7 (PMC11218294; doi:10.1186/s12957-024-03458-7)

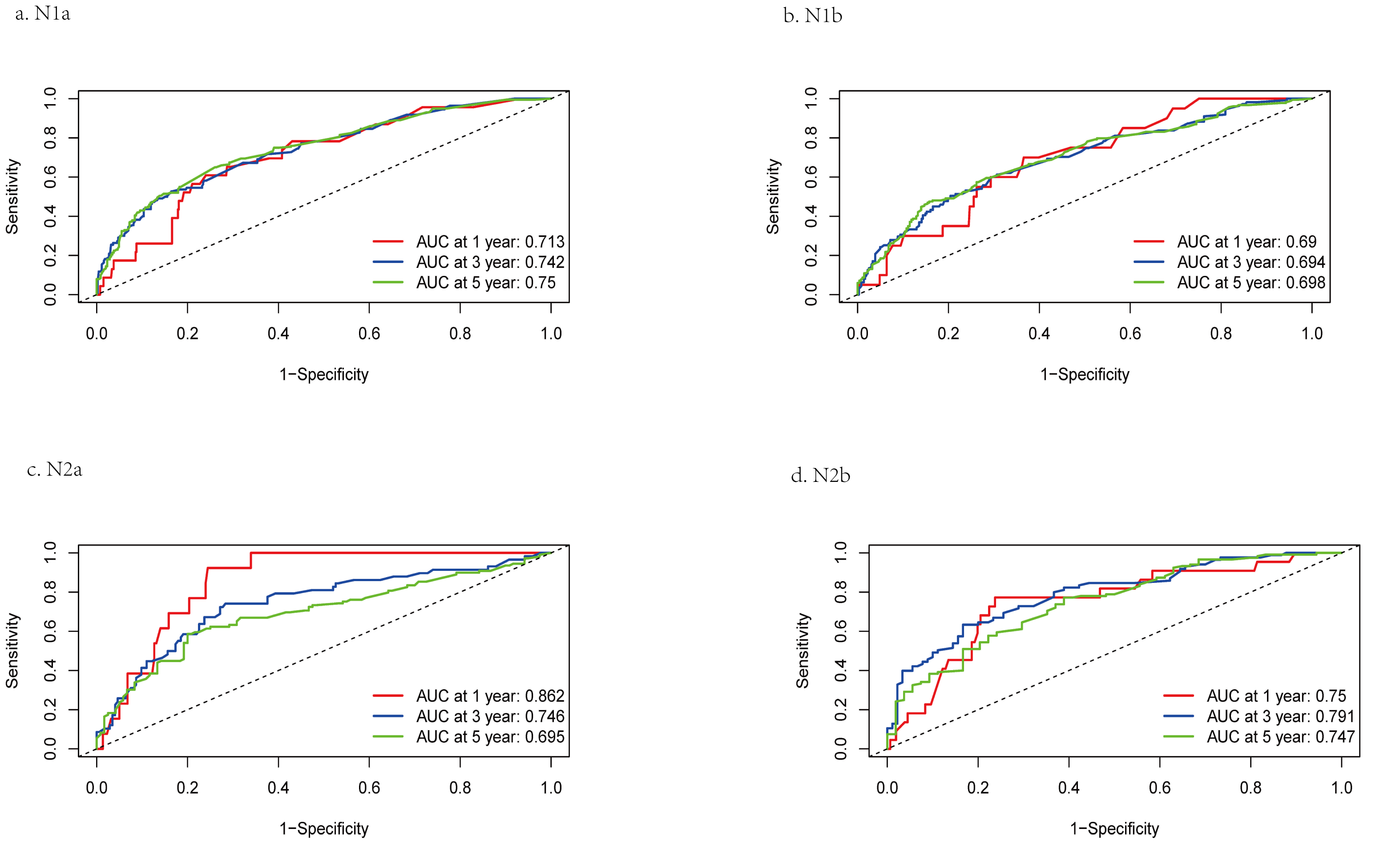

Supplement: Supplementary file 1 — Supplementary Material 1 [file 12957_2024_3458_MOESM1_ESM.tif]

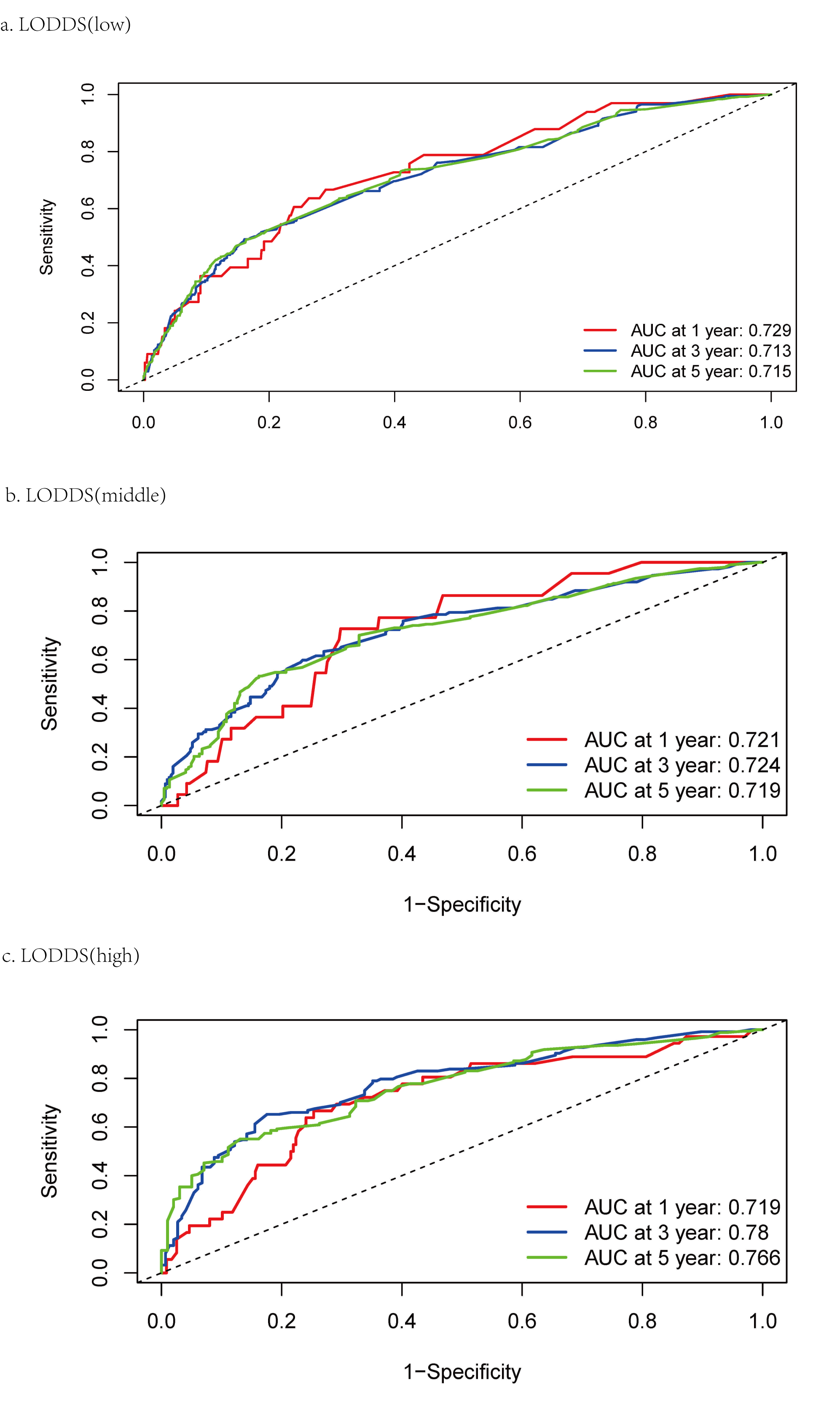

Supplement: Supplementary file 2 — Supplementary Material 2 [file 12957_2024_3458_MOESM2_ESM.tif]
