# Supplementary material for: The survival prediction of advanced colorectal cancer received neoadjuvant therapy—a study of SEER database
Source: World J Surg Oncol. 2024 Jul 1;22:175. doi: 10.1186/s12957-024-03458-7 (PMC11218294; doi:10.1186/s12957-024-03458-7)
Supplement: Supplementary file 3 — Supplementary Material 3 [file 12957_2024_3458_MOESM3_ESM.docx]

Table S1 The results of stepwise multivariate cox regression of univariate cox regression.

|  | Univariate cox regression | | Multivariate cox regression | |
| --- | --- | --- | --- | --- |
| Characteristics | HR (95%CI) | P | HR (95%CI) | P |
| **Age** |  |  |  |  |
| <50 years | reference |  |  |  |
| ≥50 years | 1.33(1.08, 1.65) | **0.008** | 1.34(1.08,1.65) | **0.008** |
| **Race** |  |  |  |  |
| white | reference |  |  |  |
| black | 1.31(0.97,1.79) | 0.082 |  |  |
| other | 1.06(0.84,1.34) | 0.597 |  |  |
| **Sex** |  |  |  |  |
| male | reference |  |  |  |
| female | 0.86(0.72,1.02) | 0.081 |  |  |
| **Marital** |  |  |  |  |
| married | reference |  |  |  |
| Unmarried +single | 1.13(0.91,1.41) | 0.266 |  |  |
| other | 1.25(1.02,1.55) | 0.034 |  |  |
| **History** |  |  |  |  |
| No | reference |  |  |  |
| Yes | 1.06(0.86,1.30) | 0.574 |  |  |
| **Location** |  |  |  |  |
| right colon | reference |  |  |  |
| left colon | 0.59(0.43,0.80) | **0.001** |  |  |
| rectum | 0.32(0.24,0.41) | **<0.001** |  |  |
| **Grade** |  |  |  |  |
| well | reference |  |  |  |
| moderately | 0.89(0.61,1.29) | 0.545 |  |  |
| poorly+ undifferentiation | 1.03(0.69,1.54) | 0.877 |  |  |
| unknown | 0.68(0.43,1.07) | 0.095 |  |  |
| **T** |  |  |  |  |
| T_X-2_ | reference |  |  |  |
| T3 | 1.35(1,1.82) | 0.047 |  |  |
| T4 | 2.2(1.57,3.08) | **<0.001** |  |  |
| **N** |  |  |  |  |
| N1 | reference |  |  |  |
| N2 | 1.5(1.25,1.79) | **<0.001** |  |  |
| **M** |  |  |  |  |
| M0 | reference |  |  |  |
| M1 | 3.26(2.74,3.87) | **<0.001** | 3.23(2.36,4.43) | **<0.001** |
| **Stage** |  |  |  |  |
| Ⅲ | reference |  |  |  |
| Ⅳ | 3.26(2.74,3.87) | **<0.001** |  |  |
| **Radiation sequence** |  |  |  |  |
| both | reference |  |  |  |
| before of surgery | 1.21(0.68,2.15) | 0.514 |  |  |
|  | Univariate cox regression | | Multivariate cox regression | |
| Characteristics | HR (95%CI) | P | HR (95%CI) | P |
| after of surgery | 2.31(1.09,4.88) | **0.028** |  |  |
| unknown | 3.38(1.87,6.08) | **0.000** |  |  |
| **Radiation** |  |  |  |  |
| Yes | reference |  |  |  |
| No +unknown | 2.75(2.28,3.32) | **<0.001** | 1.45(1.13,1.86) | **0.003** |
| **Chemotherapy** |  |  |  |  |
| Yes | reference |  |  |  |
| No +unknown | 2.28(1.34,3.87) | **0.002** | 2.60(1.51,4.47) | **0.001** |
| **Time of diagnosis to treatment** |  |  |  |  |
| ≤1 month | reference |  |  |  |
| >1 month | 1.17(1.03,1.33) | **0.018** |  |  |
| **CEA** |  |  |  |  |
| positive | reference |  |  |  |
| negative | 0.56(0.46,0.68) | **<0.001** | 0.79(0.64,0.97) | **0.024** |
| unknown | 0.75(0.61,0.93) | **0.010** | 0.89(0.71,0.11) | 0.293 |
| **Perineural invasion** |  |  |  |  |
| positive | reference |  |  |  |
| negative | 0.54(0.44,0.65) | **<0.001** | 0.67(0.55,0.82) | **<0.001** |
| unknown | 0.50(0.36,0.70) | **<0.001** | 0.66(0.47,0.92) | 0.015 |
| **Size** |  |  |  |  |
| ≤4cm | reference |  |  |  |
| >4cm | 1.42(1.18,1.70) | **<0.001** | 1.34(1.11,1.61) | **0.002** |
| unknown | 1.28(0.98,1.67) | 0.071 | 1.23(0.94,1.61) | 0.137 |
| **Examined nodes** |  |  |  |  |
| ≥15 | reference |  |  |  |
| <15 | 1.09(0.92,1.28) | 0.335 |  |  |
| **LODDS** |  |  |  |  |
| low | reference |  |  |  |
| middle | 1.14(0.93,1.40) | 0.204 | 1.16(0.94,1.42) | 0.165 |
| high | 2.09(1.70,2.58) | **<0.001** | 1.90(1.54,2.35) | **<0.001** |
| **LNR** |  |  |  |  |
| low |  |  |  |  |
| middle | 1.14(0.95,1.38) | 0.157 |  |  |
| high | 2.21(1.77,2.77) | **<0.001** |  |  |
| **Bone metastasis** |  |  |  |  |
| Yes | reference |  |  |  |
| No | 0.16(0.06,0.38) | **<0.001** |  |  |
| **Liver metastasis** |  |  |  |  |
| Yes | reference |  |  |  |
| No | 0.38(0.32,0.46) | **<0.001** | 0.63(0.49,0.81) | **0.007** |
| **Lung metastasis** |  |  |  |  |
| Yes | reference |  |  |  |
| No | 0.33(0.23,0.47) | **<0.001** |  |  |

Table S2 The results of stepwise multivariate cox regression of random forest survival regression.

| Characteristics | HR (95%CI) | P |
| --- | --- | --- |
| **M** |  |  |
| M0 | reference |  |
| M1 | 3.65(2.69,4.94) | **<0.001** |
| **Stage** |  |  |
| Ⅲ |  |  |
| Ⅳ | --- |  |
| **Chemotherapy** |  |  |
| Yes | reference |  |
| No +unknown | 2.56(1.49,4.39) | **0.001** |
| **Perineural invasion** |  |  |
| positive | reference |  |
| negative | 0.69(0.56,0.83) | **<0.001** |
| unknown | 0.66(0.47,0.92) | **0.014** |
| **Location** |  |  |
| right colon | reference |  |
| left colon | 0.73(0.53,0.99) | **0.048** |
| rectum | 0.60(0.44,0.81) | **0.001** |
| **Radiation sequence** |  |  |
| both |  |  |
| before of surgery | --- |  |
| after of surgery | --- |  |
| unknown | --- |  |
| **LNR** |  |  |
| low | reference |  |
| middle | 1.14(0.95,1.38) | **0.156** |
| high | 1.95(1.55,2.46) | **<0.001** |
| **Liver metastasis** |  |  |
| Yes | reference |  |
| No | 0.50(0.40,0.62) | **0.013** |
| **Lung metastasis** |  |  |
| Yes |  |  |
| No | --- |  |
| **LODDS** |  |  |
| low |  |  |
| middle | --- |  |
| high | --- |  |

*Note: ---: The variables were not included in stepwise multivariate analysis.*

Table S3 The results of stepwise multivariate cox regression of lasso regression.

| Characteristics | HR (95%CI) | P |
| --- | --- | --- |
| **Location** |  |  |
| right colon | reference |  |
| left colon | 0.71(0.52,0.98) | **0.036** |
| rectum | 0.59(0.43,0.80) | **0.001** |
| **T** |  |  |
| T_X-2_ | reference |  |
| T3 | 1.24(0.92,1.67) | 0.163 |
| T4 | 1.54(1.09,2.18) | **0.014** |
| **M** |  |  |
| M0 | reference |  |
| M1 | 2.56(2.09,3.14) | **<0.001** |
| **Stage** |  |  |
| Ⅲ |  |  |
| Ⅳ | --- |  |
| **Radiation sequence** |  |  |
| both |  |  |
| before of surgery | --- |  |
| after of surgery | --- |  |
| unknown | --- |  |
| **Perineural invasion** |  |  |
| positive | reference |  |
| negative | 0.72(0.59,0.88) | **0.001** |
| unknown | 0.66(0.47,0.92) | **0.016** |
| **LODDS** |  |  |
| low |  |  |
| middle | --- |  |
| high | --- |  |
| **LNR** |  |  |
| low | reference |  |
| middle | 1.15(0.96,1.39) | 0.131 |
| high | 1.98(1.57,2.48) | **<0.001** |

*Note: ---: The variables were not included in stepwise multivariate analysis.*
